# On the regulation of human D‐aspartate oxidase

**DOI:** 10.1002/pro.4802

**Published:** 2023-10-20

**Authors:** Valentina Rabattoni, Zoraide Motta, Matteo Miceli, Gianluca Molla, Alex Fissore, Salvatore Adinolfi, Loredano Pollegioni, Silvia Sacchi

**Affiliations:** ^1^ “The Protein Factory 2.0”, Dipartimento di Biotecnologie e Scienze della Vita Università degli studi dell'Insubria Varese Italy; ^2^ Dipartimento di Scienza e Tecnologia del Farmaco Università di Torino Torino Italy

**Keywords:** D‐aspartate, flavooxidase, neurotransmission, pLG72, post‐translational modification, protein–protein interaction

## Abstract

The human flavoenzyme D‐aspartate oxidase (hDASPO) controls the level of D‐aspartate in the brain, a molecule acting as an agonist of NMDA receptors and modulator of AMPA and mGlu5 receptors. hDASPO‐induced D‐aspartate degradation prevents age‐dependent deterioration of brain functions and is related to psychiatric disorders such as schizophrenia and autism. Notwithstanding this crucial role, less is known about hDASPO regulation. Here, we report that hDASPO is nitrosylated *in vitro*, while no evidence of sulfhydration and phosphorylation is apparent: nitrosylation affects the activity of the human flavoenzyme to a limited extent. Furthermore, hDASPO interacts with the primate‐specific protein pLG72 (a well‐known negative chaperone of D‐amino acid oxidase, the enzyme deputed to D‐serine degradation in the human brain), yielding a ~114 kDa complex, with a micromolar dissociation constant, promoting the flavoenzyme inactivation. At the cellular level, pLG72 and hDASPO generate a cytosolic complex: the expression of pLG72 negatively affects the hDASPO level by reducing its half‐life. We propose that pLG72 binding may represent a protective mechanism aimed at avoiding cytotoxicity due to H_2_O_2_ produced by the hDASPO enzymatic degradation of D‐aspartate, especially before the final targeting to peroxisomes.

## 1 INTRODUCTION

Since the free form of D‐aspartate (D‐Asp) was detected in several organisms (Kera et al., 1995), it has received increased attention due to the established role in the mammalian endocrine, neuroendocrine and, above all, central nervous systems (Errico et al., 2012; Errico, Mothet, & Usiello, 2015; Katane & Homma, 2011). In the brain D‐Asp not only modulates the activation state of the N‐methyl‐D‐aspartate receptors (NMDAR), by interacting with the glutamate site of the GluN2A‐D subunits (Errico, Nisticò, et al., 2008; Errico, Nisticò, Napolitano, Mazzola, et al., 2011; Errico, Nisticò, Napolitano, Oliva, et al., 2011; Errico, Rossi, et al., 2008; Olverman et al., 1988), but it also acts as an endogenous agonist of the metabotropic glutamate receptor mGluR5 (Molinaro et al., 2010). Furthermore, it was shown to trigger the release of glutamate in specific brain regions (Cristino et al., 2015; Sacchi, Novellis, et al., 2017) through the activation of presynaptic α‐amino‐3‐hydroxy‐5‐methyl‐4‐isoxazolepropionic acid receptor, NMDAR, and mGluR5.

D‐Asp shows a transient occurrence in the brain: the high levels (even exceeding those of the L‐enantiomer) detected during embryonic life drop soon after birth (Punzo et al., 2016; Sakai et al., 1998; Wolosker et al., 2000). This variation is due to the concomitant increase in the expression of D‐aspartate oxidase (DASPO or DDO, EC 1.4.3.1) (Katane & Homma, 2010; Pollegioni et al., 2021; Punzo et al., 2016), the only enzyme deputed to D‐Asp degradation in mammals (on the other hand, the enzyme responsible for D‐Asp synthesis is still unknown). Throughout the postnatal development and in the adult state, D‐Asp is maintained at low, and tightly controlled, levels by DASPO catabolic activity. Prolonged exposure to excessive non‐physiological D‐Asp concentrations in DASPO‐deficient rodent models was reported to accelerate several age‐dependent brain deterioration processes, possibly through the overstimulation of NMDAR (Cristino et al., 2015; Nuzzo et al., 2019), suggesting that DASPO plays a neuroprotective role in adult mammals (Nuzzo et al., 2019). Further studies highlighted the potential involvement of dysfunctions in D‐Asp metabolism in psychiatric disorders, such as schizophrenia (Errico, D'Argenio, et al., 2015; Nuzzo et al., 2019).

The flavoenzyme DASPO catalyzes the enantioselective oxidative deamination of acidic D‐amino acids to give the corresponding α‐keto acids and ammonia. Hydrogen peroxide is produced by the concomitant reoxidation of the reduced flavin adenine dinucleotide (FAD) cofactor by molecular oxygen (Pollegioni et al., 2021). On the other hand, neutral and basic D‐amino acids are deaminated by D‐amino acid oxidase (DAAO, EC 1.4.3.3), which is responsible for the catabolism of the NMDAR coagonist D‐serine (D‐Ser) in the brain (Pollegioni & Sacchi, 2010; Pollegioni, Sacchi, & Murtas, 2018). The human DASPO and DAAO (hDASPO and hDAAO, respectively) share the same chemical mechanism of catalysis (i.e., hydride transfer), high sequence identity, and overall tertiary structure (Kawazoe et al., 2006; Molla et al., 2020). Anyway, they differ in substrate specificity, kinetic efficiency (hDASPO shows on D‐Asp a specific activity 10‐fold higher than hDAAO on D‐Ser), oligomeric state (a stable homodimeric vs a monomeric state for hDAAO and hDASPO, respectively) and cofactor binding affinity (weak in hDAAO and strong in hDASPO) (Molla et al., 2020; Murtas et al., 2017).

The UniProtKB database reports three different isoforms of hDASPO (identifier Q99489): the canonical isoform 1 (hDASPO_341, 341 amino acids); isoform 2 (hDASPO_282), lacking residues 95–153 in hDASPO_341; and isoform 3 (hDASPO_369), harboring 28 additional N‐terminal residues, probably due to the recognition of an upstream alternative start codon. Intriguingly, the latter protein isoform has been currently identified in the hippocampus of female Alzheimer's disease patients only (Rabattoni et al., 2021). The functional properties of hDASPO_369, as well as the degradation kinetics and the mechanisms involved in protein turnover, are similar to hDASPO_341: both protein variants are active, highly stable, and mainly degraded through the ubiquitin–proteasome system (Rabattoni et al., 2021).

Here, combining *in vitro* and cellular studies, we addressed the issue of the hDASPO main isoform (hDASPO_341) regulation by post‐translational modifications (PTMs) and the binding of pLG72, the main known protein modulator of hDAAO (Murtas et al., 2022; Pollegioni, Piubelli, et al., 2018).

## 2 RESULTS

### 2.1 The “editable” cysteines in hDASPO

**FIGURE 1:**
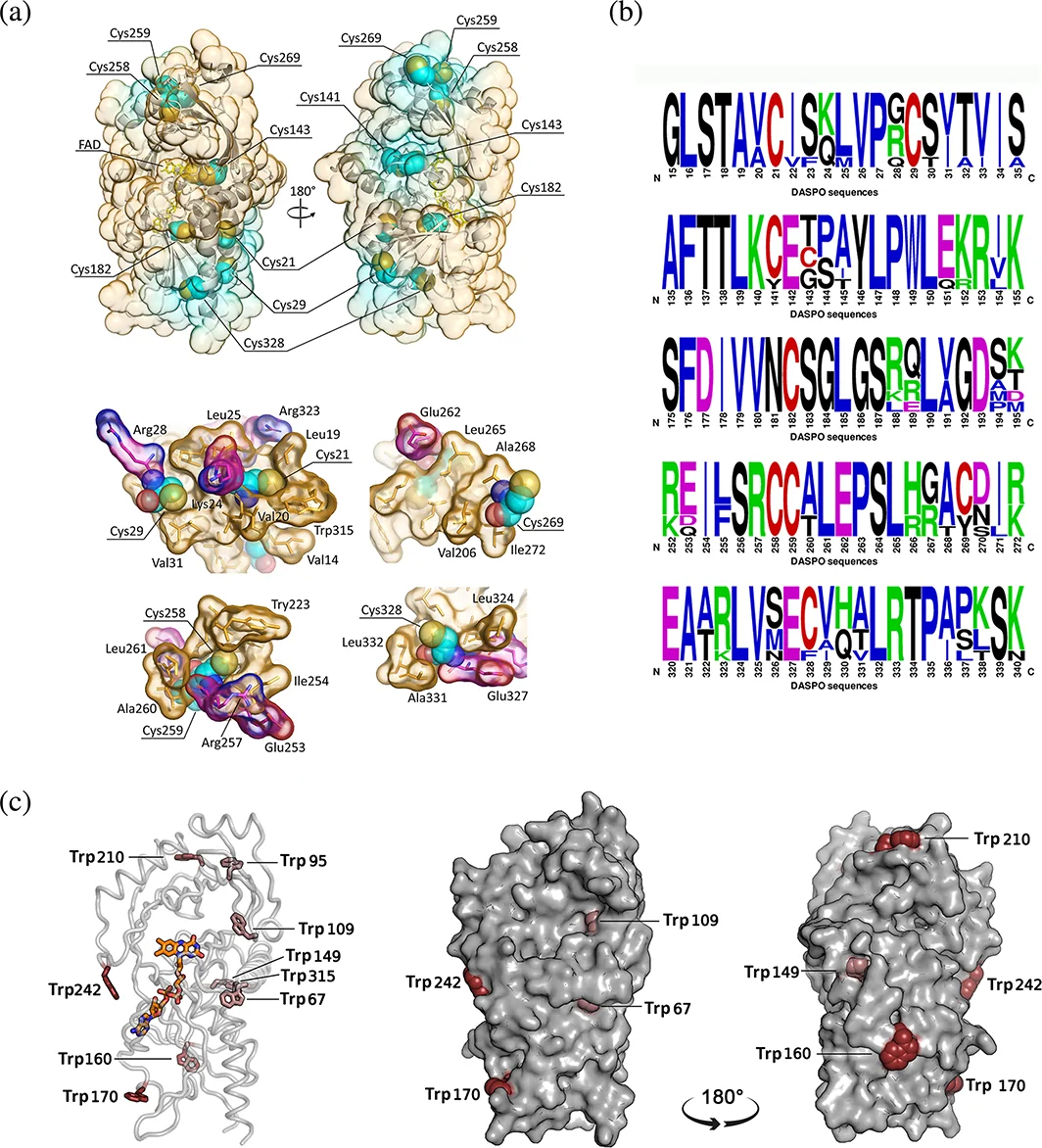
*In silico* analysis of cysteine and tryptophan residues in hDASPO. (a) Position, exposure to the solvent and amino acidic environment of cysteine residues in hDASPO. (Top) Solvent accessible surface as calculated for the hDASPO monomer (pdb code 6RKF). Cysteine residues are represented as spheres (carbon atoms in cyan, sulfur atoms in yellow); the FAD cofactor is represented as sticks (yellow). The backbone is shown as a cartoon. Protein surface is colored by surface proximity with cysteine residues (cyan = cysteine residues located within 5 Å from the surface; orange = cysteine residues located at a distance higher than 15 Å from the surface). Solvent accessible surface has been calculated based on a solvent radius of 1.4 Å. (Bottom) Analysis of the environment of selected cysteine residues, represented as spheres (carbon atoms in cyan, sulfur atoms in yellow, nitrogen atoms in blue, and oxygen atoms in red): the surrounding residues are depicted as sticks and the van der Waals surface is shown. Hydrophobic residues are colored in orange, while charged residues are colored by element (carbon atoms in magenta, nitrogen atoms in blue, and oxygen atoms in red). (b) Frequency of sequence conservation among mammalian DASPOs. Weblogo representation of conserved residues identified by the alignment of selected regions of DASPO sequences from *Homo sapiens*, *Mus musculus*, *Rattus norvegicus*, *Sus scrofa*, *Bos taurus*, *Cavia porcellus*, *Macaca fascicularis*, and *Pongo abelii*. The *x*‐axis identifies the amino acid positions (the annotated numbering refers to the human enzyme) and the height of symbols is proportional to the degree of conservation of each single residue; cysteine residues are reported in red. Figure prepared using WebLogo (https://weblogo.berkeley.edu/logo.cgi). (c) Tryptophan residues in hDASPO: solvent‐exposed tryptophans (showing an exposed surface >20 Å^2^) were considered surface residues (dark brown). FAD cofactor is represented as yellow sticks. FAD, flavin adenine dinucleotide.

hDASPO harbors nine Cys (see Figure 1a). Notably, those at positions 21, 29, 182, 258, and 259 are present in all mammalian DASPOs (Figure 1b). PTMs of cysteine residues in a protein depend on both their microenvironment and solvent exposure that modulates the reagent accessibility. With the exception of Cys141, Cys143, and Cys182, hDASPO cysteines are at or close to the protein surface, but only Cys259 and Cys269 are largely solvent‐exposed (Figure 1a, top). The presence of charged residues flanking the cysteine residue has been proposed as a feature distinguishing S‐nitrosylation sites (Gould et al., 2013). Cys269 appears far from charged residues, whereas Cys328 is located at 9.0 Å from Glu327 (Figure 1a, bottom). Also, a putative acid–base motif (Arg257 and Glu253 at 6.6 Å and 10 Å, respectively) is observed close to Cys258. The partially solvent accessible Cys21 and Cys29, within the Rossmann fold motif of the cofactor binding region, are respectively located at 10.3 and 9.0 Å from Lys24, which is part of the identified nitrosylation motif consensus sequence (Table S1), and are close to Arg28 (at 11.7 Å from Cys29) and to Arg323 (at 8.8 Å from Cys21) (Figure 1a, bottom). All accessible Cys residues are surrounded by a hydrophobic environment, an additional feature that might account for further stabilization of nitrosylated cysteines (SNO‐Cys). Altogether, these analyses suggest that at least four editable cysteine residues are present in hDASPO (Cys21, Cys29, Cys259, and Cys328).

When native hDASPO was treated with 5,5′‐dithiobis(2‐nitrobenzoic acid) (DTNB), 4.2 ± 0.4 cysteine residues reacted (consistent with structural information), while under denaturing conditions (i.e., in the presence of 4 M urea) a figure of 9.3 ± 0.7 cysteines was obtained, confirming that five cysteines are not solvent‐accessible in the native hDASPO. Notably, in hDASPO apoprotein, two additional cysteines are prone to modification in the native state compared to the corresponding holoenzyme (6.3 ± 0.3).

hDASPO and the homologous flavoenzyme hDAAO share a similar tertiary fold; however, the latter is a stable homodimer, while hDASPO is a monomer in solution. Among the five conserved cysteines in hDASPO, four are also present in the homologous flavoenzyme hDAAO (Table S1), which is modified *in vitro* following incubation with the nitric oxide (NO) donor S‐nitrosoglutathione (GSNO) (Sacchi et al., 2021): Cys18 and 264 in hDAAO (corresponding to the conserved Cys21 and 259 in hDASPO) are exposed on the protein surface and likely accessible to modification (Sacchi et al., 2021).

### 2.2 Recombinant hDASPO is modified by the NO donor GSNO and (to a low extent) by the H_2_S donor NaHS

**FIGURE 2:**
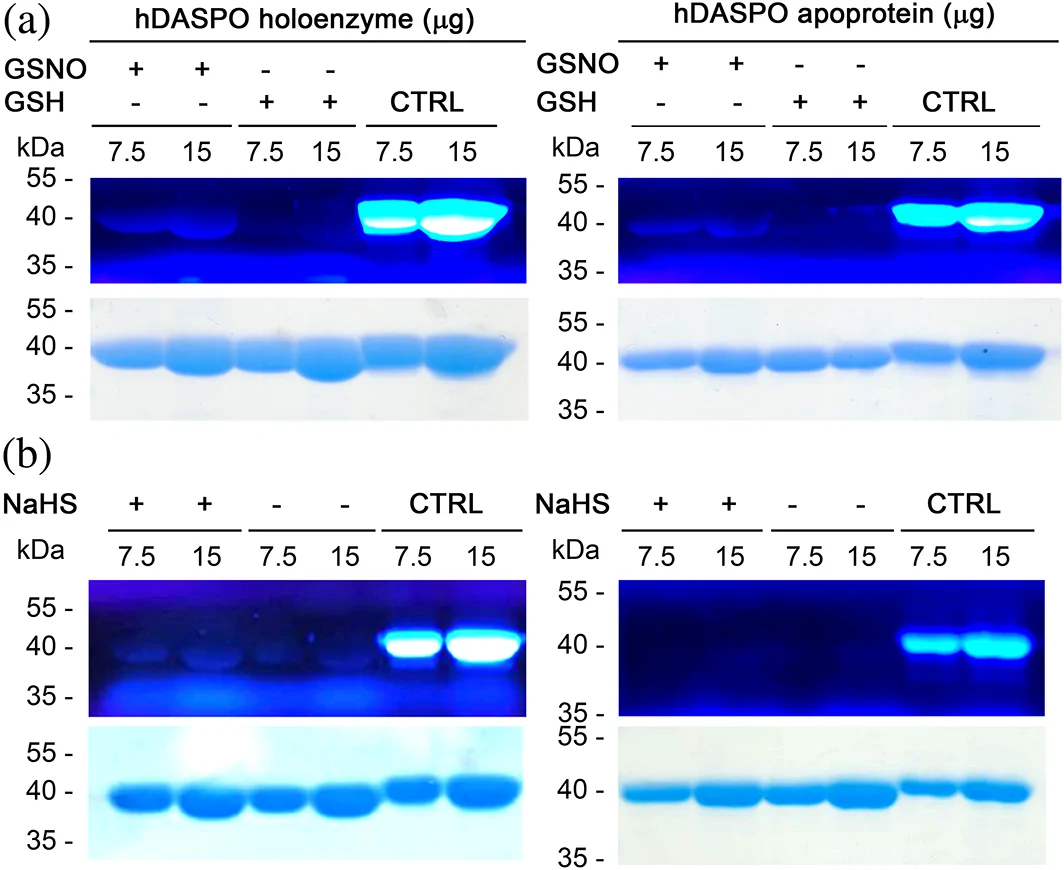
Analysis of *in vitro* S‐nitrosylation and S‐sulfhydration of hDASPO holoenzyme and apoprotein forms. Non‐reducing SDS‐PAGE analysis of recombinant hDASPO (7.5 or 15 μg) following *in vitro* S‐nitrosylation (panel a) or S‐sulfhydration (panel b). Top: fluorescence switch assay (image acquisition was performed upon excitation of the fluorescent probe). Bottom: Coomassie blue proteins staining. Mixtures in which the NO donor GSNO was replaced with GSH, or the sulfide donor NaHS was omitted were analyzed as negative controls; whereas positive controls (CTRL) were protein samples in which all cysteine residues were labeled by Alexa Fluor 350 C_5_ Maleimide (by omitting the starting blocking step during the fluorescence switch assay). GSH, reduced glutathione; GSNO, S‐nitrosoglutathione.

For sake of comparison, we investigated whether hDASPO is subjected to *in vitro* S‐nitrosylation and/or S‐sulfhydration by using the same experimental approach that has been proved effective for hDAAO (Sacchi et al., 2021). The canonical recombinant hDASPO holoenzyme or apoprotein forms (25 μM) were reacted either with 500 μM GSNO as a NO donor or 5 mM NaHS as a H_2_S donor. Negative control mixtures were prepared by adding reduced glutathione (GSH) in place of GSNO or omitting NaHS, respectively. Sample and control mixtures were analyzed by non‐reducing SDS‐PAGE and modified cysteine residues (SNO‐Cys or SH‐S‐Cys) were detected by a fluorescent switch assay (Sacchi et al., 2021). Notably, both hDASPO forms are S‐nitrosylated since a signal corresponding to Alexa Fluor 350 C_5_ maleimide labeled cysteines was apparent (Figure 2a). On the other hand, only a barely detectable signal (slightly higher compared to the control) was detected when the holoenzyme was incubated with NaHS ‐and no labeling was evident for hDASPO apoprotein (Figure 2b). The presence of bands in the negative control might be due to the presence of oxidized thiols, which do not interact with methyl methanethiosulfonate and were thus reduced during the subsequent incubation with sodium ascorbate. The latter findings suggest that unlikely hDASPO is S‐sulfhydrated, at least significantly under the conditions used.

### 2.3 Effect of nitrosylation on hDASPO properties

**FIGURE 3:**
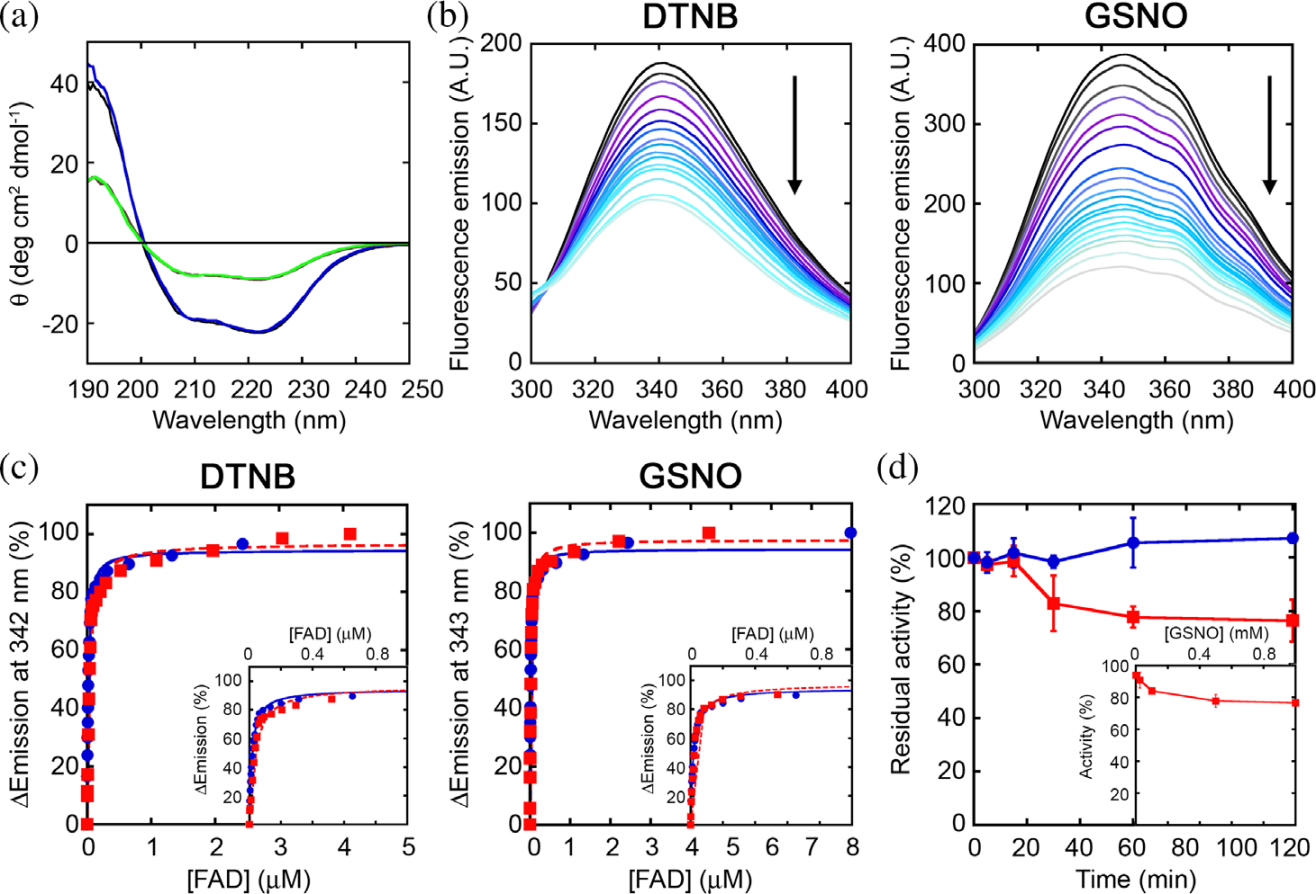
Effect of *in vitro* nitrosylation on hDASPO structural and functional properties. (a) Comparison of far‐UV CD spectra of holo‐ and apoprotein forms of hDASPO following nitrosylation (blue and green lines, respectively) with respect to controls (black and brown lines, respectively). (b) Titration of hDASPO apoprotein reacted with DTNB or GSNO with increasing amounts of FAD (the arrow indicates the spectral changes recorded at increasing cofactor concentration). (c) Analysis of FAD binding to hDASPO apoprotein following the reaction with DTNB or GSNO (as reported in panel b) assessed as quenching of protein fluorescence at ~340 nm. Values are expressed as a percentage of the total change. Blue: control mixture in the absence of DTNB or GSNO; red: mixture containing 100 μM DTNB or 500 μM GSNO. Insets show a closer view in the 0–1 μM FAD concentration range. (d) Time course of the residual activity of hDASPO in the presence (red) or absence (blue) of 500 μM GSNO at 25°C. Inset: relative activity of hDASPO after 1 hour of incubation at different GSNO concentrations (0–1 mM range). Data are the mean ± SD (*n* = 5) and are expressed relatively to the activity recorded before the incubation (=100%). DTNB, 5,5′‐dithiobis(2‐nitrobenzoic acid); GSNO, S‐nitrosoglutathione.

The effects induced by nitrosylation were studied using the recombinant hDASPO, both the apoprotein and the holoenzyme forms. As shown by circular dichroism (CD) analysis, the presence of SNO‐Cys residues did not affect the overall secondary structure either in hDASPO holoenzyme and apoprotein forms (Figure 3a). Moreover, nitrosylation did not modify the fluorescence emission spectrum of the hDASPO apoprotein, while a significant decrease (−35 ± 7.5%) in the fluorescence maximal intensity (at the emission peak at 342 nm) was apparent when hDASPO holoenzyme was reacted with GSNO (Figure S1), thus pointing to a conformational change after nitrosylation.

Since the accessible Cys21 and Cys29 are located in the cofactor binding domain, we investigated whether the induced nitrosylation perturbed the enzyme binding affinity for FAD. FAD binding to hDASPO apoprotein was studied by monitoring the quenching of apoprotein fluorescence (a total of 9 tryptophan residues are present; see Figure 1c). The protein fluorescence was unchanged by treatment with iodoacetamide, and the holoenzyme protein fluorescence was slightly modified by FAD but only by very high concentrations, not used during the titration. Nitrosylated hDASPO apoprotein was thus titrated with increasing FAD concentrations (Figure 3b). Only small changes in the dissociation constant (*K*_*d*_) values for the apoprotein–FAD complex were observed after the reaction of the apoprotein with DTNB or GSNO (3.2 ± 0.3 and 2.1 ± 0.1 × 10^−8^M, respectively; Figure 3b,c) compared to the control unreacted protein (1.6 ± 0.1 × 10^−8^M). Therefore, nitrosylation did not affect the tight interaction of the cofactor with the hDASPO apoprotein moiety.

The residual enzymatic activity at different times of incubation with the NO donor GSNO was assayed using saturating concentrations of D‐Asp (15 mM) and FAD (40 μM) at 25°C. A partial, time‐dependent inactivation was evident compared to the control mixture in which GSNO was omitted (Figure 3d): ~22% of the initial activity was lost after 1 h of incubation. Notably, a similar effect was reported for hDAAO; in that case, a protective effect by exogenous FAD was evident, in line with the weaker apoprotein‐cofactor interaction (Sacchi et al., 2021). The observed hDASPO inactivation by GSNO is concentration‐dependent, with the maximum effect measured at ≥500 μM of nitrosylating agent (Figure 3d, inset).

Cellular studies were performed on the U87 human glioblastoma cell line ectopically stably expressing hDASPO treated with the NO donors 3‐(2‐hydroxy‐1‐methyl‐2‐nitrosohydrazino)‐N‐methyl‐1‐propanamine (NOC‐7), (±)‐(E)‐4‐ethyl‐2‐[(E)‐hydroxyimino]‐5‐nitro‐3‐hexenamide (NOR‐3) or GSNO (50 μM) for 2 h. No changes in the enzyme activity were observed in samples from treated cells compared to controls (Figure S2a), suggesting that at the cellular level, the selected modifying agents failed in affecting hDASPO activity, differently to what was observed for hDAAO (Sacchi et al., 2021).

### 2.4 Effect of sulfhydration on hDASPO properties

**FIGURE 4:**
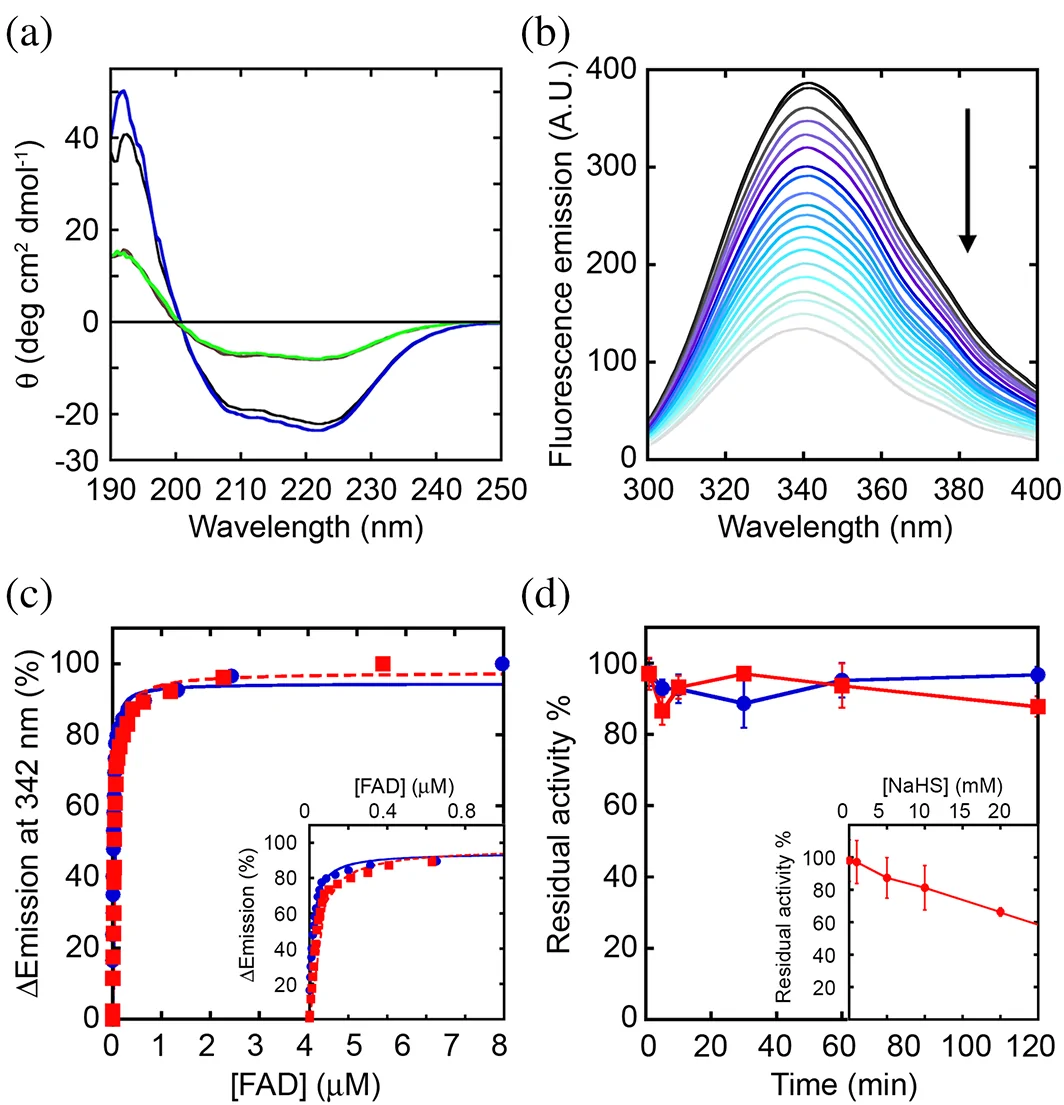
Effect of *in vitro* sulfhydration on hDASPO structural and functional properties. (a) Far‐UV CD spectra of holo‐ and apoprotein forms of hDASPO in the presence of 5 mM NaHS (blue and green lines, respectively) compared to controls (black and brown lines, respectively). (b, c) Titration of 5 μM hDASPO apoprotein with increasing amounts of FAD after sulfhydration by 5 mM NaHS: (b) spectral changes (the arrow indicates the changes recorded at increasing cofactor concentration); (c) fluorescence intensity values at 342 nm expressed as percentage of the total change. Blue: control mixture without the sulfide donor; red: mixture containing 5 mM NaHS. Inset shows a closer view in the 0–1 μM FAD concentration range. (d) Effect of sulfhydration on hDASPO residual activity at 25 °C. Blue: control; red: mixture containing 5 mM NaHS. Inset: relative activity of hDASPO after 1 h of incubation at different concentrations of NaHS. Data are the mean ± SD (*n* = 3–6) and are expressed relative to the activity measured without NaHS (=100%).

The effects induced by sulfhydration on hDASPO were studied *in vitro*: CD analysis of hDASPO holoenzyme and apoprotein showed that the overall protein structure was unaffected by incubation with 5 mM NaHS at 37°C (Figure 4a): the content in secondary structure elements remains largely unchanged as indicated by DichroWeb (http://dichroweb.cryst.bbk.ac.uk/html/home.shtml; Miles et al., 2022). Similarly, following NaHS treatment, the holoenzyme and the apoprotein fluorescence emission spectra were unchanged, as well as the *K*_*d*_ value for the apoprotein–FAD complex (3.4 ± 0.2 × 10^−8^M; Figure 4b,c).

Furthermore, NaHS did not affect the time course of hDASPO inactivation compared to the control mixture (Figure 4d), while a stabilizing effect was reported for hDAAO (Sacchi et al., 2021). A significant effect on hDASPO functionality was observed only at a high concentration of the H_2_S donor: in this condition, a concentration‐dependent inactivation was apparent and the residual activity decreased to a figure of ~65% at 20 mM NaHS (Figure 4d, inset). Anyway, such a NaHS concentration represents a non‐physiological level (as compared to 50–160 μM in the brain) (Abe & Kimura, 1996). No change in hDASPO activity was also observed at the cellular level, when U87 cells ectopically expressing the flavoenzyme were treated for 30 min with NaHS (50 and 100 μM; Figure S2b).

### 2.5 Recombinant hDASPO is not modified by PKA and PKC

Since hDASPO was predicted to be phosphorylated, likely by protein kinases A and C (PKA and PKC), among other kinases (see Table S2), *in vitro* phosphorylation experiments were carried out by incubating the purified recombinant hDASPO with three commercial kinases: PKA, PKC‐α, and PKC‐ε. The Pro‐Q Diamond phosphoprotein gel staining following SDS‐PAGE of kinase‐treated samples detected the phosphorylation of the positive controls, but not of hDASPO (Figure S3), suggesting that *in vitro* the flavoenzyme is not phosphorylated by either PKA or PKC.

### 2.6 hDASPO interaction with pLG72

We then wondered whether the interaction with putative regulatory proteins could represent an alternative mechanism of hDASPO regulation. In past years, we demonstrated that the homologous enzyme hDAAO, which is present in solution as a homodimer, interacts with the primate‐specific protein pLG72 (18 kDa) (Chumakov et al., 2002): both the wild‐type (R30) pLG72 and the Arg30Lys variant (R30K, related to schizophrenia susceptibility) (Sacchi et al., 2008; Sacchi, Cappelletti, et al., 2017) bind to hDAAO (80 kDa) yielding a ~200 kDa protein complex, and act as negative chaperone (by inactivating the enzyme and pushing its cellular turnover) thus preventing an excessive degradation of D‐Ser and avoiding cytosolic H_2_O_2_ accumulation (Cappelletti et al., 2014; Sacchi et al., 2016).

**FIGURE 5:**
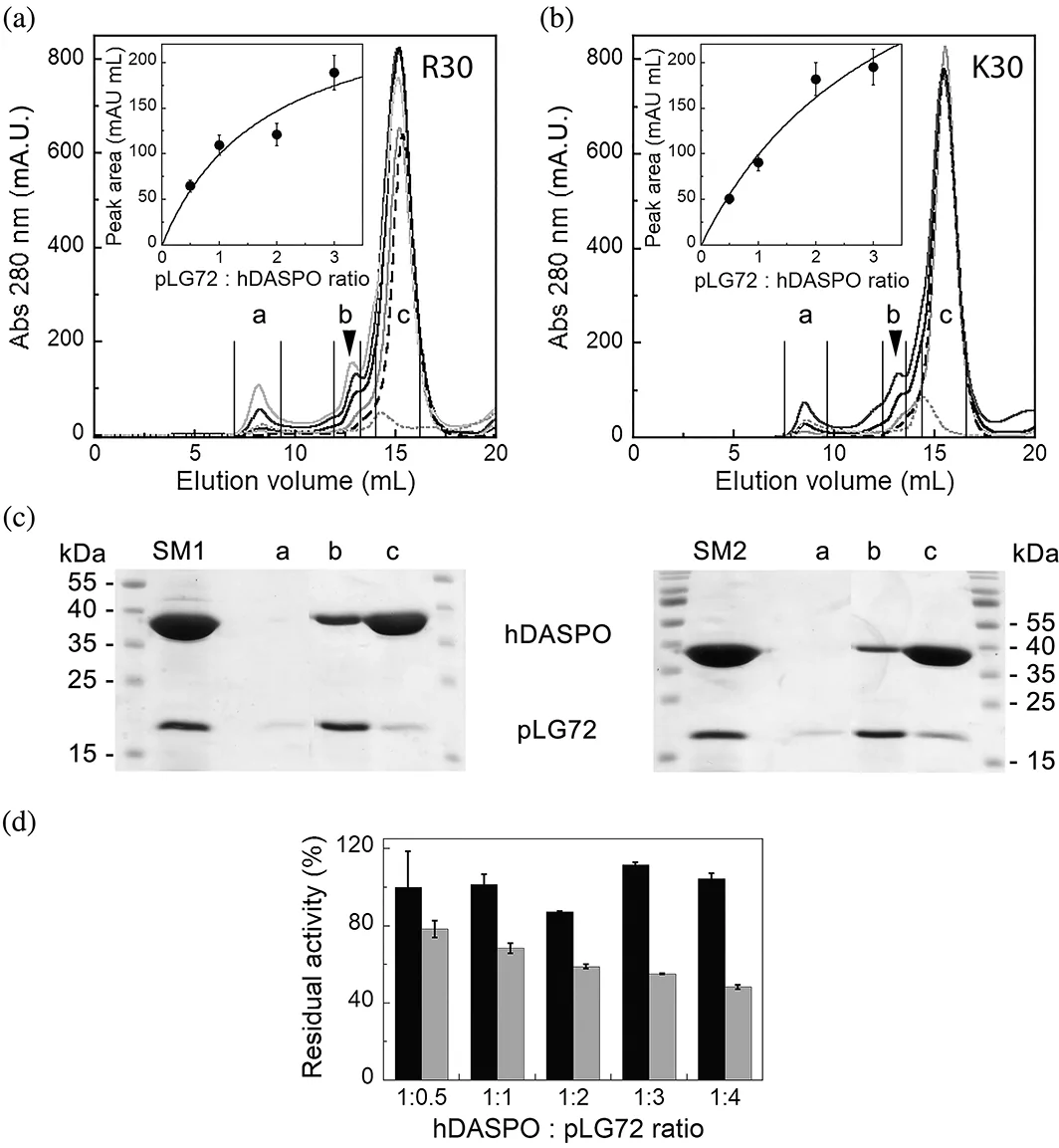
Effect on hDASPO properties by binding of pLG72 variants. (a, b) SEC analysis of hDASPO binding to R30 (a) and R30K (b) pLG72 variants. Protein mixtures containing 25 nmol of hDASPO and different amounts of pLG72 variants corresponding to 0.5–3 molar ratios were incubated at 4 °C for 10 min and centrifuged before column injection. Dotted curves: 25 nmol hDASPO; dashed curves: 25 nmol pLG72. The recorded elution profiles showed multiple peaks corresponding to: hDASPO (at ~15.5 mL), pLG72 (at ~8.0 and ~14.2 mL), and hDASPO–pLG72 protein complex (at 13.0 ± 0.2 mL). Insets: effect of hDASPO:pLG72 molar ratio on the area of the 13 mL peak (~114 kDa), corresponding to the hDASPO complexes with the R30 and R30K pLG72 variants, and indicated by the arrowheads in the chromatograms. (c) SDS‐PAGE analysis of the SEC fractions. SM: sample mixtures containing hDASPO (25 nmoles) and R30 or R30K  pLG72 variants (50 nmoles). Bands corresponding to hDASPO (15 μg) and pLG72 variants (7 μg) were detected at 39 and 18 kDa, respectively. About 30 μL of the indicated SEC fractions (a–c) was also analyzed. (d) Effect on hDASPO activity of pLG72 variants. Sample mixtures were prepared by adding a fixed amount of recombinant hDASPO (0.4 nmol) with increasing amounts of R30 (black bars) or R30K (gray bars) pLG72 variants and incubated for 30 min at 25 °C. hDASPO residual activity was reported as percentage with respect to the initial value. Data are reported as mean ± SE (*n* = 3).

Recombinant hDASPO holoenzyme is monomeric in solution (Molla et al., 2020): its solubility and oligomerization were not affected by the presence of 0.06% N‐lauroyl sarcosine (NLS), a detergent needed to maintain pLG72 in a monodispersed form (Sacchi et al., 2008). Therefore, hDASPO:pLG72 interaction was investigated by titrating a fixed amount of hDASPO with increasing amounts of the R30 or R30K pLG72 variants. The size exclusion chromatography (SEC) elution profile showed the appearance of a peak (at 13 mL, ~114 kDa), distinct from those corresponding to the un‐complexed hDASPO and pLG72 (elution volume of 15.5 and 14.4 mL, respectively, Figure 5a,b). As revealed by SDS‐PAGE analysis (Figure 5c), the 13 mL peak fraction contained the two proteins either using the R30 or the R30K pLG72 variants. In both cases, the area of the peak at 114 kDa increased to the amount of pLG72 present in the mixture (Figure 5a,b, insets). Based on the observed saturation curves and the elution volume in SEC analysis, we assume the generation of a complex made by 2 pLG72 (18 × 2 kDa) and 2 hDASPO monomers (39.2 × 2 kDa), and that the two pLG72 variants bind the flavoenzyme with an apparent similar avidity. *In silico* predicted models of the hDASPO–pLG72 complex are reported in Figure S4.

This interaction was further investigated by microscale thermophoresis (MST). At first, hDASPO was reacted with the RED‐NHS fluorescent dye, then a fixed amount of the labeled enzyme (10 nM) was incubated with increasing concentrations (0.003–100 μM) of the label‐free pLG72 variants. Based on the variation of the fluorescence signal, the fraction of the labeled hDASPO bound to the R30 or R30K pLG72 variants is indicative of a complex formation (Figure S4a). For both the pLG72 variants, the amplitude of the variation in the detected signal showed a reverse sigmoidal trend (Figure S4). The *K*_*d*_ values were similar and in the μM range (6.8 ± 0.6 and 3.8 ± 0.4 μM for R30 and R30K pLG72, respectively), thus suggesting a weak interaction with hDASPO, similar to the hDAAO:pLG72 complex (Sacchi, Cappelletti, et al., 2017).

Intriguingly, the R30 and R30K pLG72 variants differentially affect hDASPO functionality (Figure 5d): no change in the enzymatic activity was observed for the wild‐type R30 pLG72 (even at a high molar ratio), while a significant decrease in hDASPO activity by the R30K pLG72 variant was apparent at the highest molar ratios, after an incubation of 30 min at 25 °C.

### 2.7 Cellular studies confirm the hDASPO–pLG72 interaction

Immunofluorescence studies were used to substantiate hDASPO–pLG72 interaction at the cellular level. At different times following transient transfection in both U87 cells (control) and in U87 cells stably expressing R30K pLG72 tagged with a C‐terminal ECFP, three distinct distribution patterns for hDASPO were apparent: i) homogeneously diffused into the cytosol; ii) localized both in sub‐cellular structures (previously demonstrated to be peroxisomes) (Rabattoni et al., 2021) and (largely) in the cytosol; iii) completely compartmentalized in peroxisomes (Figure S6a,b). A very similar distribution was previously reported for hDAAO (Murtas et al., 2019; Sacchi et al., 2011). In control U87 cells, the distribution pattern differs over time after transfection: at 24 h, hDASPO is mostly cytosolic in a higher number of cells, while at 72 h, it is predominantly present within the peroxisomes. Intriguingly, at 24 h after transfection, compartmentalization of hDASPO was twofold higher in U87 cells overexpressing pLG72 compared to control cells (65% and 32%, respectively; Figure S6b). This observation might indicate that the hDASPO targeting process was faster, or rather that the cytosolic levels of the flavoenzyme were lower when pLG72 was simultaneously overexpressed. Western blot analysis supports the latter hypothesis: in the cells expressing pLG72, substantially lower amounts of the flavoenzyme were observed compared to U87 control cells at all times after transfection (Figure S6c,d). In particular, hDASPO expression was barely detectable at 24 h, suggesting that the presence of pLG72 hampered the flavoenzyme accumulation, likely by increasing its degradation rate. Notably, we previously reported that hDASPO is a long‐lived protein in U87 cells (*t*_1/2_ ~ 100 h) (Rabattoni et al., 2021).

When transfected cells were treated with cycloheximide (CHX) to block protein synthesis at 48 h after transfection, the levels of hDASPO were strongly decreased only in U87 cells also expressing pLG72 (Figure S7), yielding to a half‐life value of ~10 h. On the other hand, and in line with the marked cellular stability of the tagged flavoenzyme (Rabattoni et al., 2021), no significant change in hDASPO levels was detected in U87 control cells: a trend to hDASPO accumulation was apparent (Figure S7). These findings strongly support a regulatory role exerted by pLG72 on hDASPO cellular stability.

**FIGURE 6:**
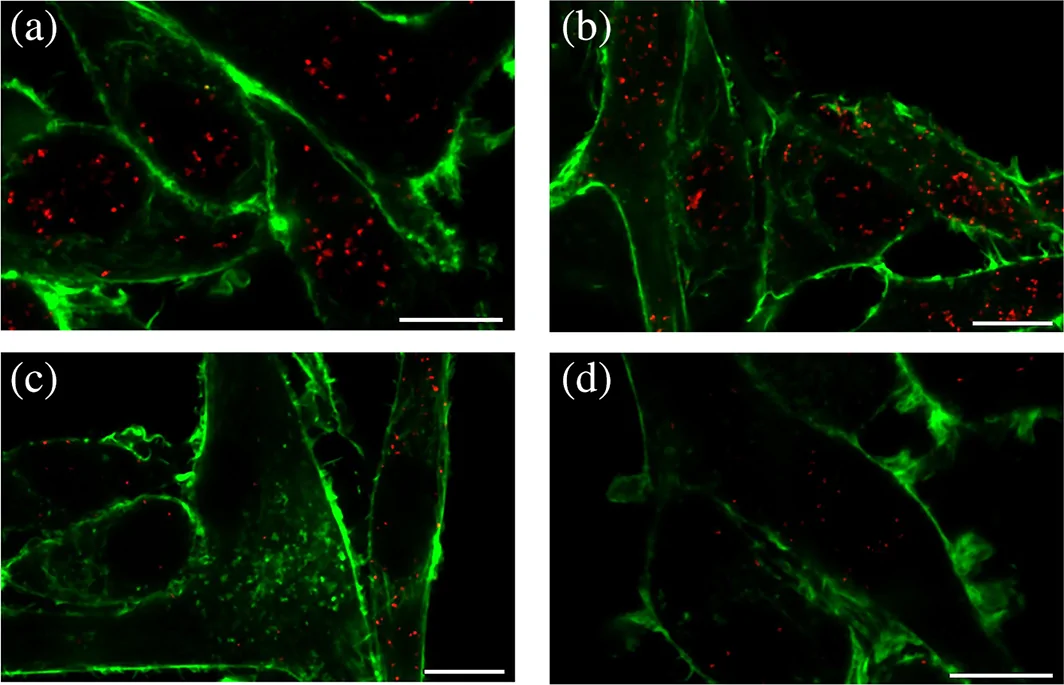
Investigation of hDASPO–pLG72 interaction at the cellular level. (a–c) U87 cells expressing pLG72‐ECFP were transiently transfected with a plasmid encoding hDASPO and were fixed at 24 (a), 48 (b), and 72 h (c) after transfection. The hDASPO–pLG72 interaction was detected via the Duolink PLA technique using mouse anti‐FLAG and rabbit anti‐pLG72 antibodies. (d) Negative control was prepared by performing the assay on non‐transfected U87 cells. Red: proximity spots indicating protein interaction. Green: cytoskeleton stained with Phalloidin CruzFluor™ 488. Scale bar = 10 μm.

Finally, a proximity ligation assay (PLA) was performed on the same U87 cells to further investigate hDASPO:pLG72 interaction at the cellular level. In this case, a signal indicative of the close proximity (< 40 nm) of the target proteins was detected by confocal microscopy. Notably, spots of proximity were detected in U87 cells expressing pLG72 after the transfection with hDASPO (Figure 6a–c) and not in the negative control (U87 untransfected cells incubated with the same antibodies, Figure 6d), suggesting that the two proteins are actually interacting. The density of the detected spots changes after transfection: at 24 and 72 h less than 10 PLA signals/cell were evident in most of the transfected cells (70%), whereas very few cells (1.25% and 0%, respectively) contained 30 or more spots. The higher signal density was observed at 48 h in 33% of the transfected cells (Figure 6b).

## 3 DISCUSSION

A study on *post‐mortem* brain samples of schizophrenic patients reported a substantial decrease in D‐Asp content, associated with a significant increase in hDASPO activity, with no variation in the levels of its encoding transcript (Nuzzo et al., 2017). These findings suggest that the enzyme's function should be modulated *in vivo* by different mechanisms (e.g., PTMs and/or the interaction with effector molecules).

S‐nitrosylation is a reversible, non‐enzymatic reaction wherein NO is covalently attached to a thiol group of a protein cysteine residue to form an S‐nitrosothiol. Noteworthy, it has been reported that in U87 cells, nitrosylation regulates the activity of both serine racemase (SR) and hDAAO (Shoji et al., 2006), the main enzymes involved in D‐Ser metabolism: NO was shown to inhibit SR while enhancing hDAAO activity. Most recently, human SR was reported to follow a biphasic inhibition process by S‐nitrosylation with a fast phase associated with the modification of Cys113; Cys128 and Cys269 were also modified (Marchesani et al., 2018). *In vitro* nitrosylation experiments and cellular studies on U87 cells ectopically expressing hDAAO treated with different NO donors reported a partial but significant inactivation of the flavoenzyme (Sacchi et al., 2021). Based on a similar catabolic role in the brain and overall tertiary structure, here we used the same experimental approach to investigate whether this PTM could regulate hDASPO activity. *In vitro* analyses were performed on the recombinant canonical hDASPO_341, which represents the main isoform and the only one that can be currently produced in large amounts in a soluble form. It contains nine cysteines, of which four are highly conserved in mammalian DASPOs and DAAOs (Figure 1). All cysteines are likely present in the free reduced form and are on the solvent‐accessible surface, with the exception of Cys141, Cys143, and Cys182 (Figure 1a). Consistent with structural information, experimental data indicate that four cysteines are easily accessible in the native hDASPO holoenzyme and potentially exposed to modifications (namely Cys21, Cys29, Cys259, and Cys328), while two additional residues are exposed in the apoprotein form. Cys21 and Cys29 are close to two charged residues (Arg28 and Arg323; Figure 1a), a feature that has been proposed to distinguish S‐nitrosylation sites (Gould et al., 2013), and are within the Rossman fold motif involved in flavin binding. However, despite *in vitro* experiments confirming that recombinant hDASPO can be nitrosylated (Figure 2a,b), spectral and functional experiments suggested that the enzyme's properties were only marginally affected by this modification. These findings were confirmed at the cellular level. Actually, no difference in the enzymatic activity was observed in U87 cells ectopically expressing hDASPO when treated with different NO donors (Figure S2a), indicating that the flavoenzyme is not nitrosylated to a significant extent at the cellular level under the experimental condition used and that hDASPO and hDAAO activities are differently modulated (notably, the latter is largely present in the apoprotein form at physiological concentrations of FAD and D‐Ser, a relaxed conformation more prone to modifications compared to hDASPO). In the case of hDASPO, the apparent discrepancy with the *in vitro* results may be due to specific control mechanisms (i.e., the interaction with regulatory proteins could prevent protein modification).

Hydrogen sulfide (H_2_S) has emerged as an important gasotransmitter and signaling molecule (Paul & Snyder, 2015): the corresponding modification, that is, sulfhydration, occurs on reactive Cys residues like nitrosylation does. When hDASPO was incubated with a sulfide precursor (NaHS), only barely detectable levels of S‐sulfhydration were observed, coupled to a partial inactivation (66% residual activity) at high NaHS concentrations only (i.e., 20 mM), representing a not physiological condition (Abe & Kimura, 1996).

Bioinformatic analyses assigned a high prediction score for phosphorylation to several residues; in particular, hDASPO is putatively subjected to phosphorylation by PKA, PKC, and other protein kinases involved in the cell cycle regulation (cdc2 and CKII; Table S2). However, the recombinant enzyme was not modified *in vitro* by PKA and PKC (Figure S3). Further studies are needed to verify whether the enzyme is phosphorylated in human tissues.

Concerning regulation by protein interaction, hDAAO is specifically regulated by the binding of the small, primate‐specific protein pLG72; see Murtas et al. (2022) for a recent review. Similarly, both the wild‐type protein and the pathological R30K variant bind hDASPO with micromolar affinity, yielding the formation of a ~114 kDa complex. The binding of pLG72 R30K to hDASPO brings to its partial inactivation without hampering the tight interaction with the FAD cofactor. Interestingly, and unlike hDAAO, hDASPO activity was not affected by pLG72 R30 binding. In U87 cells stably expressing pLG72‐ECFP and transiently expressing hDASPO, PLA analysis showed that pLG72 (likely exposed on the outer membrane of the mitochondria) (Sacchi et al., 2008, 2016) interacts with hDASPO. Actually, in pLG72‐ECFP expressing cells, lower levels of hDASPO were detected compared to control ones, in particular at 24 h after transfection, indicating that pLG72 negatively affects hDASPO cellular stability by decreasing its half‐life.

## 4 CONCLUSIONS

hDASPO is prone to nitrosylation *in vitro*, a modification yielding to moderate inactivation. No consistent evidence for sulfhydration or phosphorylation was instead revealed. hDASPO interacts with pLG72, a process affecting the cellular level of the flavoenzyme. Considering that pLG72 is assumed to act as a negative chaperone of hDAAO, boosting its degradation (Murtas et al., 2022; Sacchi et al., 2016) and, consistently with the observed decrease in hDASPO cellular levels and half‐life when pLG72 is simultaneously expressed, we propose that pLG72 plays a similar role on hDASPO. Since the latter is an extremely active and efficient flavoenzyme (Molla et al., 2020), the presence of a significant amount of the substrate might produce an abnormal increase in the ROS species H_2_O_2_ and, eventually, induce a stress condition. The hDASPO:pLG72 complex formation might represent a safety mechanism to avoid such an insult, especially for the enzyme fraction present in the cytosol before being targeted to peroxisomes. Overall, this represents the first study providing an insight into the regulation of hDASPO, the main enzyme controlling D‐Asp availability in human tissues. Further studies are needed to assess whether specific cellular signals or metabolic alterations, such as an abnormal increase in D‐Asp levels or an oxidative stress process, may induce the modulation of the enzymatic activity through PTMs or interacting partners.

## 5 MATERIALS AND METHODS

### 5.1 Recombinant protein expression and purification

Recombinant hDASPO, pLG72 R30, and R30K variants were produced in *Escherichia coli* and purified as stated in Molla et al. (2020) and Molla et al. (2006), respectively. Briefly, the recombinant hDASPO encoded by the pET11‐His‐hDASPO plasmid was produced as soluble protein in the *E. coli* BL21(DE3) LOBSTR strain (Merck Biosciences, Darmstadt, Germany), and the recombinant protein was purified from the crude cell extract on a HiTrap chelating column (GE Healthcare, Chicago, IL, USA). hDASPO apoprotein was prepared weakening the binding of the FAD cofactor by extensive dialysis against halide anions (1.5–3 M KBr) and a mild denaturant (1 M urea) at pH 8.0, as reported in Molla et al. (2020). On the other hand, R30 and R30K pLG72 variants were expressed upon induction in *E. coli* BL21‐Codon plus (DE3)‐RIL cells (Stratagene, San Diego, CA, USA) and recovered from insoluble inclusion bodies obtained following cell lysis and centrifugation, using the procedure detailed in Molla et al. (2006).

### 5.2 Molecular modeling and structural analyses

Solvent‐accessible surface area analysis in hDASPO (PDB entry code 6RKF) was performed using the algorithm provided by the Pymol open‐source software (https://pymol.org/2/) as recently reported in Sacchi et al. (2021). This parameter is defined as the surface traced out by the center of a water sphere, having a 1.4 Å radius rolled over the protein atoms.

### 5.3 Determination of accessible cysteines

The number of accessible cysteines in hDASPO holoenzyme and apoprotein was evaluated experimentally by reacting the two protein forms with 100 μM DTNB as reported for hDAAO (Sacchi et al., 2021).

### 5.4 *In vitro* S‐nitrosylation

The reducing agent present in the recombinant hDASPO preparation was eliminated as detailed in Sacchi et al. (2021). The specific activity of hDASPO in the absence of any reducing agent was identical to the value in the presence of 2‐mercaptoethanol. *In vitro* nitrosylation was performed by incubating the recombinant hDASPO (both the holo‐ and the apoprotein forms) with GSNO as a NO donor (Sigma‐Aldrich, St. Louis, MO, USA) as reported in Sacchi et al. (2021). Mixtures in which GSNO was replaced by 500 μM reduced glutathione (Sigma‐Aldrich) were prepared as negative controls. All reaction mixtures were incubated for 1 h in the dark, at 25 °C, under constant rotation. S‐Nitrosylation was verified by fluorescence detection, following a fluorescence switch assay and gel electrophoresis analysis as detailed in Sacchi et al. (2021). Positive controls were obtained by omitting the methyl methanethiosulfonate blocking step, that is, all cysteines in hDASPO were reduced during the subsequent incubation with sodium ascorbate.

### 5.5 *In vitro* sulfhydration

The modification of cysteine residues in hDASPO was investigated by incubating the recombinant protein with NaHS (Santa Cruz Biotechnology, Dallas, TX, USA) as a hydrogen sulfide donor. Recombinant hDASPO samples (diluted in not reducing buffer to a final concentration of 1 mg/mL) were added of 5 mM NaHS and incubated at 37 °C for 1 h. The presence of modified cysteines was analyzed by the fluorescence switch assay following non‐reducing SDS‐PAGE.

### 5.6 Spectral measurements

Fluorescence spectra were recorded at 15 °C using a Jasco FP‐750 instrument (Jasco Europe Srl, Cremella, Italy) on hDASPO apoprotein samples reacted with 100 μM DTNB, or 500 μM GSNO, or 5 mM NaHS (as reported above), diluted to a final concentration of 1 μM (0.04 mg/mL) in 100 mM sodium pyrophosphate, pH 8.0; spectra were corrected for the buffer contribution (excitation at 280 nm). The binding constant for the cofactor was determined by titrating the apoprotein with increasing amounts of FAD and following the quenching of the protein fluorescence at ~340 nm (Molla et al., 2020):

(1) $$K_{d}=\frac{\left(\left[P\right]\left[L\right]\right)}{\left[\mathrm{PL}\right]},$$

where [P], [L], and [PL] represent molar concentrations of the protein, ligand, and complex, respectively. To subtract the contribution of flavin, blank measurements were performed on buffer solution added with FAD at the same concentrations used during the titration.

CD far‐UV spectra were recorded using a Jasco J‐815 spectropolarimeter (Jasco Europe Srl) after *in vitro* nitrosylation or sulfhydration at a final protein concentration of 0.1 mg/mL in water.

### 5.7 Activity assay

Following *in vitro* nitrosylation or sulfhydration reactions (0–1 mM or 0–20 mM for GSNO and NaHS, respectively), the enzymatic activity of recombinant hDASPO was assayed polarographically using an oxygen electrode (Molla et al., 2020). Aliquots of the reaction mixtures were diluted 1:20 in not reducing buffer and immediately assayed for hDASPO activity at pH 8.5, air saturation, and 25 °C, using 15 mM D‐Asp as a substrate, in the presence of 40 μM FAD (i.e., at a saturating concentration of both the substrate and the cofactor). Control measurements were performed by omitting the NO or H_2_S donors. Variation between groups was evaluated by two‐way analysis of variance with the Bonferroni post hoc test for multiple comparisons.

The effect of pLG72 variants on hDASPO stability was evaluated by assaying the flavoenzyme activity on mixtures containing the two proteins at different molar ratios: a fixed amount of hDASPO (0.4 nmol) was added with increasing amounts of the R30 or R30K pLG72 variants (0.2–1.6 nmol) in storage buffer (20 mM Tris–HCl, pH 8.5, 150 mM NaCl, 5% glycerol, 5 mM 2‐mercaptoethanol, 40 μM FAD and 0.06% NLS). The different mixtures were incubated at 25 °C for 30 min and hDASPO residual activity was determined as above and compared with those determined in control mixtures lacking pLG72.

### 5.8 Size exclusion chromatography

The evaluation of the hDASPO–pLG72 complex formation was performed by SEC using a Superdex 200 column (GE Healthcare, Chicago, IL, USA) and storage buffer. Sample mixtures were prepared by adding different amounts (12.5–75 nmol) of R30 or R30K pLG72 variants to 25 nmol of hDASPO, incubated at 4 °C for 10 min and centrifuged before loading. Single protein solutions (25 nmol of hDASPO or pLG72) were also analyzed as controls. Elution profiles were recorded following the absorbance at 280 nm, and fractions corresponding to the different peaks were collected and subjected to SDS‐PAGE analysis.

### 5.9 Microscale thermophoresis

The binding affinity between hDASPO and the pLG72 variants was determined by MST using the Monolith NT.115 instrument (NanoTemper Technologies GmbH, München, Germany). About 0.1 nmol of hDASPO was labeled using the RED‐NHS labeling kit second generation (MO‐L011, NanoTemper Technologies) following the recommended procedure by the manufacturer. The dye carries a reactive NHS‐ester group that covalently binds to primary amines (lysine residues). The final concentration of hDASPO‐RED was ~3 μM (as calculated from its absorbance at 280 nm, corrected by the absorbance of the dye) and the degree of labeling was ~0.7. Binding mixtures were set up by adding 10 nM hDASPO‐RED to solutions at different concentrations (3 nM to 100 μM) of the pLG72 R30 and R30K variants.

### 5.10 Cellular studies

Human U87 glioblastoma cells (ATCC, Manassas, VA, USA) ectopically expressing the canonical hDASPO isoform (341 amino acids) (Rabattoni et al., 2021) were maintained in Dulbecco's modified Eagle medium supplemented with 10% fetal bovine serum, 1 mM sodium pyruvate, 2 mM L‐glutamine, 1% penicillin/streptomycin, and 2.5 μg/mL amphotericin B (all from Euroclone, Pero, Italy) at 37 °C in a 5% CO_2_ incubator.

To evaluate the effect of either nitrosylation or sulfhydration on hDASPO functional properties at the cellular levels, U87 cells ectopically expressing the flavoenzyme were treated by adding to the growth medium 50 μM of the NO donors NOR‐3, NOC7 (Santa Cruz Biotechnology), or GSNO for 2 h, or 50 and 100 μM of NaHS for 30 min (Sacchi et al., 2021). Control experiments were set up by adding an equal amount of the vehicle (dimethylsulfoxide or H_2_O). The hDASPO enzymatic activity was assayed on cell lysates using the Amplex UltraRed reagent (Thermo Fisher Scientific, Monza, Italy) (Rabattoni et al., 2021).

To assess the role of pLG72 in controlling hDASPO degradation, control U87 cells, and U87 cells stably expressing pLG72‐ECFP (Cappelletti et al., 2014) were transfected with the pcDNA3‐3xFLAG‐hDASPO 341 construct by using the FuGENE HD transfection reagent (Promega, Madison, WI, USA). Forty‐eight hours after transfection, cells were treated with CHX (100 μg/mL) to block protein synthesis. At different times after CHX addition, cell lysates were analyzed by Western blot analyses for the detection of hDASPO levels.

### 5.11 Immunofluorescence analysis and in situ proximity ligation assay

hDASPO cellular localization in U87 cells ectopically expressing pLG72‐ECFP and in control cells was investigated by immunofluorescence and confocal analysis (Rabattoni et al., 2021). The cells were seeded on gelatin‐coated coverslips (5 × 10^4^ cells each), transfected with the pcDNA3‐3xFLAG‐hDASPO 341 construct and fixed at different times after transfection (24, 48, and 72 h). Detection was carried out by a mouse monoclonal anti‐FLAG antibody (MA1‐91878, Thermo Fisher Scientific; diluted 1:500) and, after extensive washing, a donkey anti‐mouse AlexaFluor 546 antibody (Invitrogen; diluted 1:1000). Nuclei were counterstained with the far‐red fluorescent nuclear probe DraQ5 (Thermo Fisher Scientific; diluted 1:500 in phosphate‐buffered saline).

To investigate hDASPO:pLG72 interaction at the cellular level, U87 cells expressing pLG72‐ECFP were transfected as above and, after fixation and permeabilization, subjected to PLA using the Duolink In Situ Orange starter kit mouse/rabbit (Sigma‐Aldrich), following the supplier indicated procedure (Rabattoni et al., 2023). The mouse anti‐FLAG antibody and a rabbit polyclonal anti‐pLG72 antibody (PA5‐97653, Thermo Fisher Scientific; both antibodies diluted 1:500 in Duolink antibody diluent) were used for the target protein detection. Cells were counterstained with Phalloidin CruzFluor™ 488 conjugate (Santa Cruz Biotechnology; diluted 1:1000) for 1 h at room temperature to visualize the cytoskeleton. Stained coverslips were imaged using an inverted laser scanning confocal microscope (TCS SP5, Leica Microsystems, Wetzlar, Germany) equipped with a 63.0 × 1.25 NA plan apochromatic oil immersion objective as reported in Rabattoni et al. (2021, 2023).

## AUTHOR CONTRIBUTIONS

**Valentina Rabattoni**: Formal analysis; investigation; data curation; writing—review and editing. **Zoraide Motta**: Investigation; formal analysis; writing—review and editing; data curation. **Matteo Miceli**: Investigation; data curation; writing—review and editing. **Gianluca Molla**: Formal analysis; investigation; writing—review and editing. **Alex Fissore**: Investigation; formal analysis; writing—review and editing. **Salvatore Adinolfi**: Investigation; formal analysis; writing—review and editing. **Loredano Pollegioni**: Conceptualization; supervision; resources; writing—original draft. **Silvia Sacchi**: Conceptualization; writing—original draft; investigation; formal analysis; data curation; supervision; resources.

## CONFLICT OF INTEREST STATEMENT

The authors declare no conflict of interest.

## Supporting information


**DATA S1.** *In silico* prediction analyses of putative S‐nitrosylation and phosphorylation sites (Tables S1 and S2). Additional data related to *in vitro* and cellular studies aimed at verifying the hDASPO modifications and their effect on the enzymatic activity (Figures S1–S3). Finally, *in silico* analysis and supplementary results from *in vitro* and cellular studies planned to investigate hDASPO:pLG72 interaction are reported (Figures S4–S7).
