# Supplementary material for: On the regulation of human D‐aspartate oxidase
Source: Protein Sci. 2023 Nov 1;32(11):e4802. doi: 10.1002/pro.4802 (PMC10588558; doi:10.1002/pro.4802)
Supplement: Supplementary file 1 — DATA S1. In silico prediction analyses of putative S‐nitrosylation and phosphorylation sites (Tables S1 and S2). Additional data related to in vitro and cellular studies aimed at verifying the hDASPO modifications and their effect on the enzymatic activity (Figures S1–S3). Finally, in silico analysis and supplementary results from in vitro and cellular studies planned to investigate hDASPO:pLG72 interaction are reported (Figures S4–S7). [file PRO-32-e4802-s001.docx]

**Supplementary materials**

**Suppl. Table 1: Prediction of nitrosylation sites by web-based tools.** Cysteine residues indicated in bold are strictly conserved in hDASPO and hDAAO (see Fig. 1B). In the last column, the underscored residues are those present in the consensus sequence of identified putative nitrosylation motifs by SNO site. It is noteworthy that all the prediction tools identified the vast majority of hDASPO cysteines as not modified, with the exception of Cys21, 29, 259, 328. According to the protein structure (23) all cysteine residues of hDASPO should be present in the free reduced form.

The prediction of putative S-sulfhydration sites is currently feasible only using the pCysMOD tool (44), which identified Cys21 (albeit with a high false positive rate, 13%) and 328 as potentially modified residues. Beside this, accumulated evidence suggests that the sulfhydration of cysteine residues may share similar chemical features with protein S-nitrosylation (45).

| Cys position | GPS  SNO 1.0 | pCys  MOD | Deep  Nitro | Pre  SNO | Sequence  -10 +10 |
| --- | --- | --- | --- | --- | --- |
| **21** | ● |  |  |  | AGVVGLSTAV**C**ISKLVPRCSV |
| 29 |  | ● |  |  | AVCISKLVPR**C**SVTIISDKFT |
| 141 |  |  |  |  | VFGQAFTTLK**C**ECPAYLPWLE |
| 143 |  |  |  |  | GQAFTTLKCE**C**PAYLPWLEKR |
| **182** |  |  |  |  | LHPSFDIVVN**C**SGLGSRQLAG |
| **258** |  |  |  |  | AENSREILSR**C**CALEPSLHGA |
| **259** | ● |  |  |  | ENSREILSRC**C**ALEPSLHGAC |
| 269 |  |  |  |  | CALEPSLHGA**C**NIREKVGLRP |
| 328 |  | ● |  |  | ALEAARLVSE**C**VHALRTPIPK |

GPS SNO 1.0 at <http://sno.biocuckoo.org> (46); pCysMOD at <http://pcysmod.omicsbio.info/webserver.php>; (44); DeepNitro at <http://deepnitro.renlab.org/webserver.html> (47); PreSNO at http://kurata14.bio.kyutech.ac.jp/PreSNO/.

**Suppl. Table 2: Prediction of phosphorylation sites in hDASPO by web-based tools.** For NetPhos 3.0, the prediction score value was set above 0.75. Despite several hDASPO residues have been proposed as modified by the different predictors, the estimated levels of serine, threonine and tyrosine phosphorylation are heterogeneous. However, 5 (21%) threonine, 4 (17%) serine and 1 (14%) tyrosine residues are predicted as putative phosphorylation sites by at least two predictors. Moreover, when a single kinase is specified as the putative modifying enzyme, hDASPO should be mainly subjected to phosphorylation by PKA, PKC, and protein kinases involved in cell cycle regulation (cdc2 and CKII).

| Residues (total) | NetPhos 3.1 | PhosphoSitePlus | PHOSPHONET | GPS 6.0 |
| --- | --- | --- | --- | --- |
| **Ser (23)** | 5 (22%) | 3 (9%) | 8 (35%) | 4 (17%) |
| **Thr (24)** | 9 (37%) | 2 (12%) | 10 (41%) | 2 (12%) |
| **Tyr (7)** | 1 (14%) | 0 | 6 (86%) | 0 |

NetPhos 3.1 at https://services.healthtech.dtu.dk/services/NetPhos-3.1/ (48); PhosphoSitePlus at <https://www.phosphosite.org/homeAction>; PHOSPHONET at <http://www.phosphonet.ca/>; GPS 6.0 at <https://gps.biocuckoo.cn/>


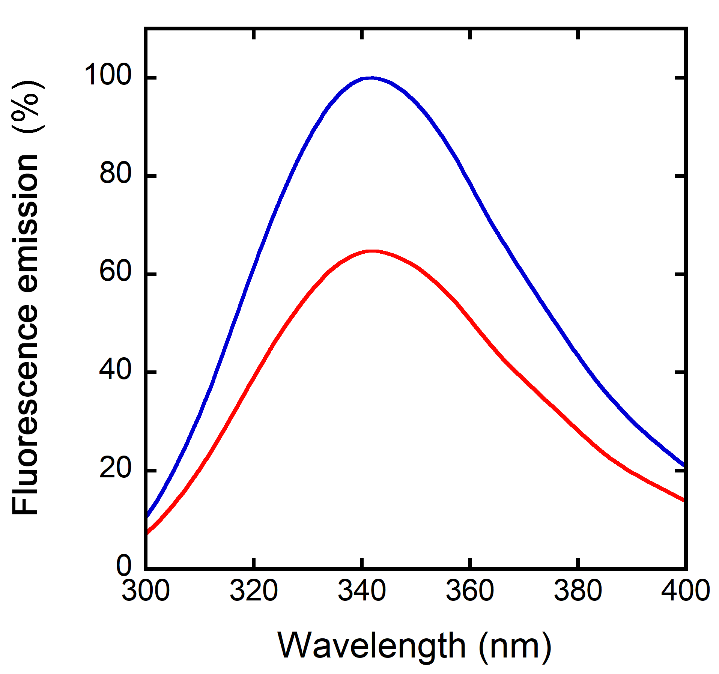


**Suppl. Fig. 1: Comparison of fluorescence spectra of hDASPO holoenzyme before and after nitrosylation.** The blue line indicates the untreated hDASPO, while the red line represents the average spectrum of the protein added of 500 µM GSNO (n=3). Values are expressed as percentage compared to the peak at 342 nm of the unreacted sample. Measurements were performed at 15° C.


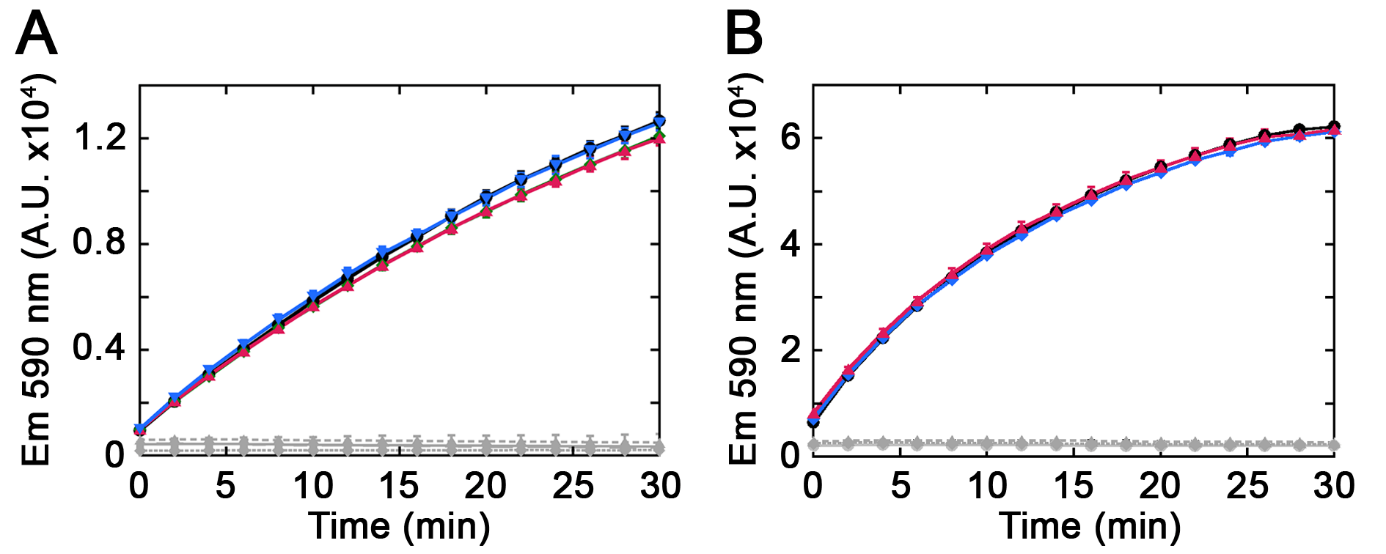


**Suppl. Fig. 2:Effect of nitrosylation and sulfhydration on hDASPO activity in the model cellular system.** (A) U87 cells stably expressing hDASPO were treated with 50 µM NOR-3 (red line), NOC-7 (blue line), GSNO (green line) or with an equal amount of DMSO (black line). No significant variation in hDASPO activity was evident between the treatments and control samples and no hDASPO activity was measured in U87 cells transfected with the empty vector and added with NOR-3 (dotted grey line), NOC-7 (dashed grey line) or DMSO (solid grey line). (B) The same cells were treated with 50 (blue line) or 100 µM NaHS (red line), or with an equal amount of DMSO (black line). Also in this case no difference between controls and treatments was observed. No signal change was recorded when control U87 cells were added with 50 µM (dotted grey line) or 100 µM NaHS (dashed grey line), or DMSO (solid grey line). The hDASPO activity was determined by the Amplex UltraRed assay; data were expressed as fluorescence change at 590 nm over time. Data are the mean ± SD (n = 4).


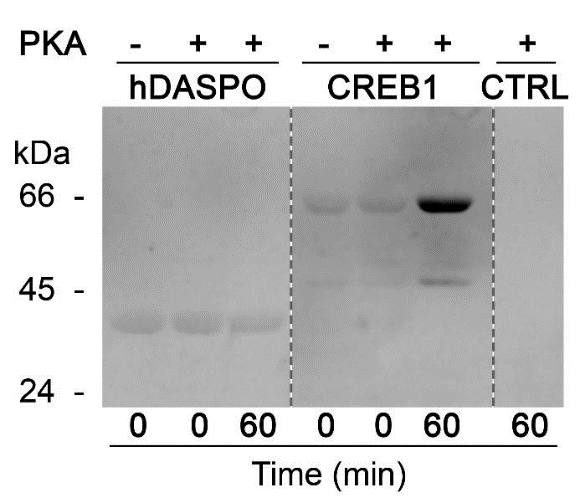


**Suppl. Fig. 3:** **SDS-PAGE analysis of recombinant hDASPO following *in vitro* phosphorylation.** At different time points (0 and 60 minutes) after the addition of PKA, aliquots of the reaction mixture corresponding to 2 µg of hDASPO were withdrawn, added with SDS-PAGE sample buffer and loaded on a 12% acrylamide gel. Mixtures in which PKA or hDASPO (CTRL) were omitted have been analyzed as negative controls (first and last lane, respectively), whereas for positive controls hDASPO was replaced by CREB1 (1.2 µg/lane). Dashed lines represent non-continuous lanes. Image acquisition was performed upon excitation of the fluorescent dye.

The PKA reaction mixture contained 3.6 µM (7 µg) of recombinant hDASPO in 50 mM Tris-HCl, pH 7.5, 10 mM MgCl2, 1 mM 1,4-dithiothreitol and 50 µM ATP, in a final volume of 50 µL. Mixtures prepared by omitting or by replacing hDASPO with 2.2 µM of the recombinant GST-tagged CREB1 (4 µg; Merck KGaA, Darmstadt, Germany) were prepared as negative and positive controls (49), respectively. Then, 9 mU of recombinant PKA (Merck) diluted in 50 mM HEPES, pH 7.4, 1 mM DTT was added to each assay mixture to start the reaction. Analogously, PKC-α and PKC-ε (Ab Cam, Cambridge, UK) reaction mixtures containing 7 µg of recombinant hDASPO in 20 mM HEPES, pH 7.4, 10 mM MgCl2, 1 mM DTT, 1 mM CaCl2, 0.5 mg/mL phosphatidylserine, 0.05 mg/mL diacylglycerol, 1 mM sodium *o*-vanadate and 50 µM ATP were set up. In these cases, positive controls used the histone H1 (3.8 µM, 4 µg; Sigma-Aldrich) (50). Then, 1 mU of the recombinant PKC-α or 0.5 mU of PKC-ε (both diluted in 65 mM Tris-HCl, pH 7.5, 0.87% NaCl (w/v), 2 mM DTT) were added to start the reaction. All reactions were performed at 30 °C and at different time points (0, 2 hours, and overnight), aliquots of the reactions (15 µL, 2 µg hDASPO) were withdrawn and blocked by adding 4X Laemmli sample buffer (5 µL) and boiled. The different samples were resolved by SDS-PAGE and phosphorylated proteins were detected by using the Pro-Q Diamond Phosphoprotein Gel Stain (Thermo Fisher Scientific, Waltham, MA, USA), following the procedure indicated by the supplier. As a staining control, a molecular ladder (PeppermintStick Phosphoprotein Molecular Weight Standards; Thermo Fisher Scientific) containing phosphorylated (45.0 and 23.6 kDa) and not phosphorylated (116, 66.2, 18.0, and 14.4 kDa) proteins was used. Gels were imaged upon acquisition with an Odyssey Fc imaging system (LI-COR Biosciences, Lincoln, NE, USA) and the 600 nm channel with a light source for excitation. A 59-kDa band corresponding to CREB1 was evident, whose intensity increased with the incubation time. No difference in the staining intensity was instead detected for hDASPO and the negative control lacking PKA. Similar results have been obtained for PKC-α and PKC-ε treated samples: a band corresponding to the histone H1 was evident upon staining for phosphoproteins while no signal was detected for hDASPO (not shown).

**hDASPO:pLG72 complex: *in silico* analysis and mode of interaction prediction**

Both pLG72 wild-type (R30) and R30K variants are homodimers in solution (34). The homodimer structure was predicted using HDOCK server (51) and the model of pLG72 R30K structure produced in (52). The model of pLG72 R30 was generated using PyMol by introducing an Arg at position 30 in the pLG72 R30K structure.

Two symmetric models of the dimer (named dimer a and b) were predicted for both pLG72 variants. Dimer a is slightly more probable than dimer b based on the HDOCK confidence score: 0.96 and 0.90 (for dimer a and b of pLG72 R30, respectively) and 0.94 and 0.87 (for dimer a and b of pLG72 R30K) (Suppl. Fig. 4A). Thus, both pLG72 variants are predicted to dimerize in the same manner (i.e., the presence of an Arg or a Lys at position 30 does not affect the dimerization of the protein). Indeed, residue 30 is buried at the dimer interface in both dimers.

The dimer models were used in docking experiments to predict interaction with hDASPO (i.e., a complex between one dimer of pLG72 and two monomers of hDASPO). The docking simulations were performed using the experimental structure of hDASPO (PDB code: 6RKF, subunit A) (23). When pLG72 dimer a was used, only one solution allowing the simultaneous interaction of two hDASPO monomers was obtained. Each hDASPO monomer interacted in a symmetrical way with the pLG72 dimer (confidence score: 0.86). As expected, pLG72 R30 and R30K produced the same results (Suppl. Fig. 4B left). When pLG72 dimer b was used to predict the complex, several solutions allowed the simultaneous interaction of two hDASPO monomers with the pLG72 dimer. Again, the solutions with the best confidence score (0.92) produced using the two pLG72 variants were superimposable (Suppl. Fig. 4B right).

Altogether, the presence of an Arg or a Lys at position 30 does not seem to affect the *in silico* predicted interaction between pLG72 and hDASPO.


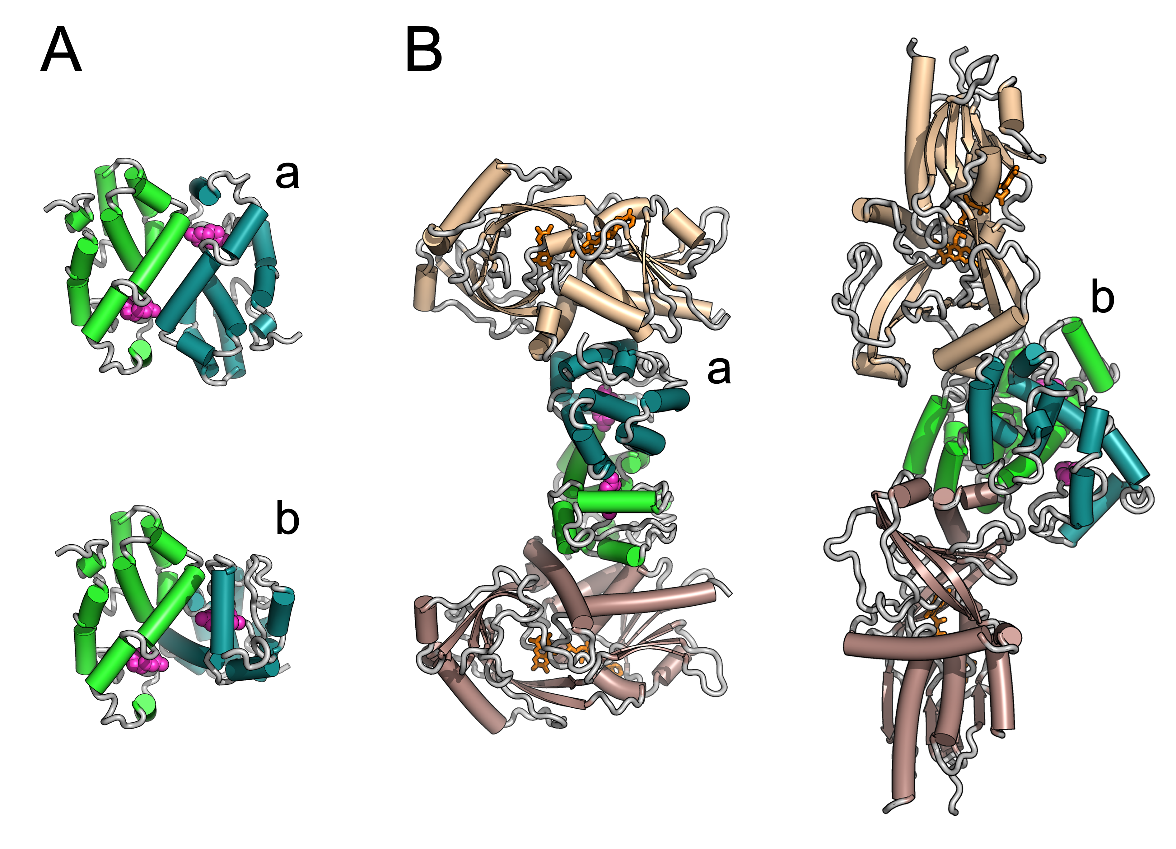


**Suppl. Fig. 4: Prediction of the interaction between one dimer of pLG72 and two monomers of hDASPO by protein-protein docking analysis.** A) Predicted models of dimerization of pLG72 (shown in green and teal). Only dimer a (top) and dimer b (bottom) of wild-type pLG72 (R30) are shown. Residue Arg30 is represented as purple spheres. B) Predicted complexes between one dimer of pLG72 R30 (dimer a, left, or b, right) and two monomers of hDASPO (shown in light and dark brown). The FAD cofactor is shown in orange.


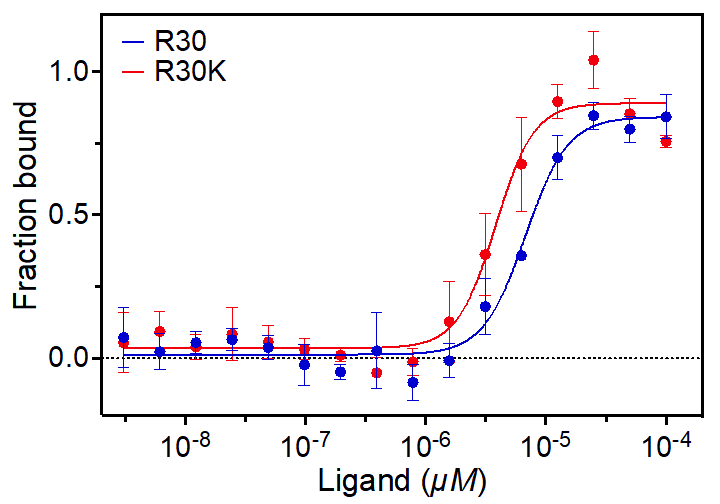


**Suppl. Fig. 5: MST analysis of hDASPO:pLG72 interaction.** hDASPO was labeled using RED-NHS 2nd Generation kit by mixing 10 μL of the 300 μM dye solution to 90 μL of 10 μM hDASPO (30 min in the dark). Labeled hDASPO was separated from dye excess using gravity gel permeation (B-column). pLG72 R30 (black line) and R30K (green line) was titrated into a fixed concentration of labeled hDASPO. The recording was performed at 20 °C using the NANO red LED at 100% excitation power and the MST power at 40%. The cold region was from -1.0 to 0 seconds and hot region from 4.0 to 5.0 seconds. The Kd values were tested using on time strategy. Fitting curves were obtained by analysis within MO.Affinity Analysis v3.0.5.


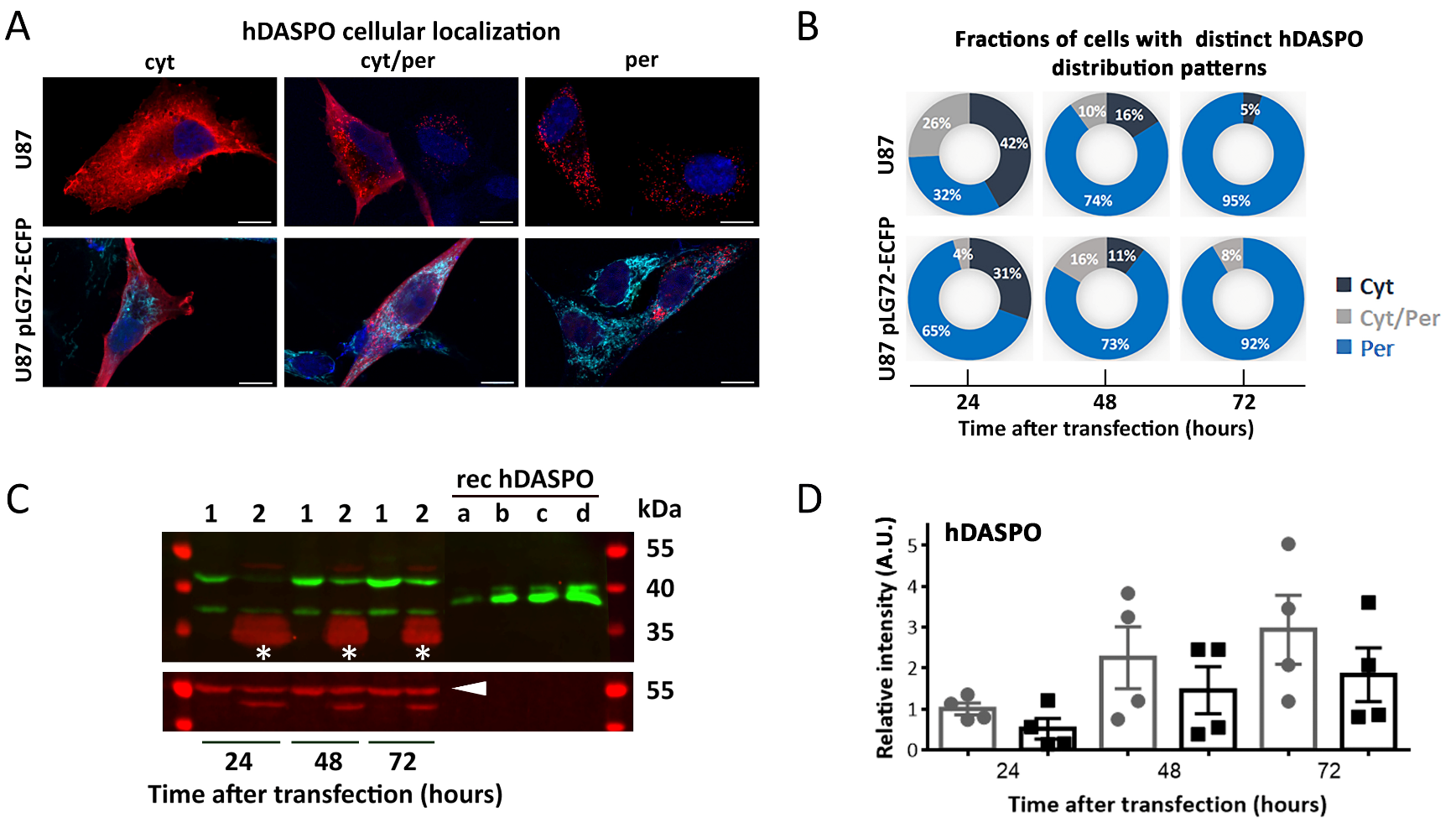


**Suppl. Fig. 6: hDASPO cellular levels and distribution in U87 transfected cells.** A) After transient transfection, hDASPO showed three different distribution patterns in both U87 control cells and in the ones ectopically expressing R30K-ECFP pLG72: cytosolic (cyt), partially localized within peroxisomes but still largely present in the cytosol (cyt/per), and completely compartmentalized into peroxisomes (per). Red: hDASPO; cyan: pLG72-ECFP; blue: nuclei counterstained with Draq5. Scale bar = 10 µm. B) Fraction of cells displaying the aforementioned patterns of hDASPO distribution at different times after transfection. Data (50-100 cells/condition) were collected manually. C) Western blot analysis comparing hDASPO expression levels in the two U87 cell lines, at different times after transfection. Top) Samples corresponding to 20 µg total proteins were loaded. Lane 1: U87 control cells; lane 2: pLG72 expressing U87 cells. Green = hDASPO; red = pLG72-ECFP (asterisk). Lanes a-d: different amounts of purified, recombinant hDASPO used as standard (2.5, 5, 7.5, and 12.5 ng). Bottom) The membrane was also analyzed using the anti--tubulin antibodies as a loading standard, to normalize signals corresponding to hDASPO and pLG72 variants. D) Densitometric analyses of the band corresponding to hDASPO, detected by Western blotting in transfected control U87 and U87 R30K-ECFP pLG72 cells (gray vs black columns). The detected signals were normalized by β-tubulin and reported relatively to U87 control cells at 24 h after transfection. Error bars represent standard error (n=4).

**Suppl. Fig. 7: Effect of CHX treatment on ectopically expressed hDASPO levels in U87 cells.** Bars correspond to the densitometric analysis of the band for hDASPO in U87 (grey bar) and U87 R30K-ECFP pLG72 (black bars) transfected cells and analyzed by Western blot at different times after treatment with CHX (100 µg/mL). The hDASPO signals were normalized to β-tubulin ones and reported using values at the time of CHX addition (0 hours) as a reference.
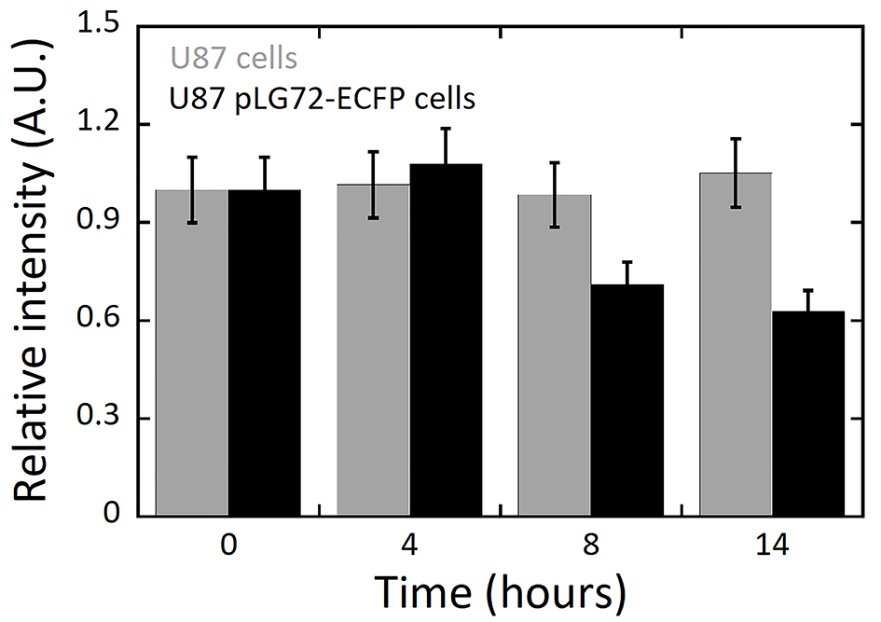


**References Supplementary materials**

1. Li S, Yu K, Wu G, Zhang Q, Wang P, Zheng J, et al (2021) pCysMod: Prediction of multiple cysteine modifications based on deep learning framework. Front Cell Dev Biol 9:617366.
2. Ju Y, Fu M, Stokes E, Wu L, Yang G (2017) H₂S-Mediated protein S-sulfhydration: A prediction for its formation and regulation. Molecules 22:1334.
3. Xue Y, Liu Z, Gao X, Jin C, Wen L, Yao X, et al (2010) GPS-SNO: computational prediction of protein S-nitrosylation sites with a modified GPS algorithm. PLoS One 5:e11290.
4. Xie Y, Luo X, Li Y, Chen L, Ma W, Huang J, et al (2018) DeepNitro: Prediction of protein nitration and nitrosylation sites by deep learning. genomics proteomics Bioinformatics. 16:294-306.
5. Blom N, Gammeltoft S, Brunak S (1999) Sequence and structure-based prediction of eukaryotic protein phosphorylation sites. J Mol Biol 294:1351-1362.
6. Gonzalez GA, Montminy MR (1989) Cyclic AMP stimulates somatostatin gene transcription by phosphorylation of CREB at serine 133. Cell 59:675-680.
7. Zhao M, Sutherland C, Wilson DP, Deng J, Macdonald JA, Walsh MP (2004) Identification of the linker histone H1 as a protein kinase C epsilon-binding protein in vascular smooth muscle. Biochem Cell Biol 82:538-546.
8. Yan Y, Tao H, He J, Huang SY (2020) The HDOCK server for integrated protein-protein docking. Nat Protoc 15:1829-1852.
9. Birolo L, Sacchi S, Smaldone G, Molla G, Leo G, Caldinelli L, Pirone L, Eliometri P, Di Gaetano S, Orefice I, Pedone E, Pucci P, Pollegioni L (2016) Regulating levels of the neuromodulator D-serine in human brain: structural insight into pLG72 and D-amino acid oxidase interaction. FEBS J 283:3353-70.
